# Supplementary material for: Magnetic eye tracking in mice
Source: eLife. 2017 Sep 5;6:e29222. doi: 10.7554/eLife.29222 (PMC5584990; doi:10.7554/eLife.29222)
Supplement: Transparent reporting form [file elife-29222-transrepform.docx]

***eLife’s* transparent reporting form**

We encourage authors to provide detailed information *within their submission* to facilitate the interpretation and replication of experiments. If you have any questions, please contact us: [editorial@elifesciences.org](mailto:editorial@elifesciences.org).

**Sample-size estimation**

- You should state whether an appropriate sample size was computed when the study was being designed
- You should state the statistical method of sample size computation and any required assumptions
- If no explicit power analysis was used, you should describe how you decided what sample (replicate) size (number) to use

Please outline where this information can be found within the submission (e.g., page numbers or figure legends), or explain why this information doesn’t apply to your submission:

Sample sizes (Figure 4E,F; Figure 5; Figure 6; Figure 7C,E,G; n = 7-9) were chosen according to standard sample sizes used previously in the lab (e.g., Kimpo et al. Elife, 2014). For Figure 4C, more mice were included (n = 24) because extra magnetic eye tracking calibration data was available. Fewer mice were used for a subset of technically difficult experiments which did not involve statistical tests (Figure 3C; Figure 7F).

**Replicates**

- You should report how often each experiment was performed
- You should include a definition of biological versus technical replication
- The data obtained should be provided and sufficient information should be provided to indicate the number of independent biological and/or technical replicates
- If you encountered any outliers, you should describe how these were handled
- Criteria for exclusion/inclusion of data should be clearly stated
- High-throughput sequence data should be uploaded before submission, with a private link for reviewers provided (these are available from both GEO and ArrayExpress)

Please outline where this information can be found within the submission (e.g., page numbers or figure legends), or explain why this information doesn’t apply to your submission:

Number of replicates and an indication of whether the replicate was technical (Figure 1) or biological (all other Figures) are indicated within the text and figure legends.

Criteria for exclusion can be found in Materials and Methods (line 467)

**Statistical reporting**

- Statistical analysis methods should be described and justified
- Raw data should be presented in figures whenever informative to do so (typically when N per group is less than 10)
- For each experiment, you should identify the statistical tests used, exact values of N, definitions of center, methods of multiple test correction, and dispersion and precision measures (e.g., mean, median, SD, SEM, confidence intervals; and, for the major substantive results, a measure of effect size (e.g., Pearson's r, Cohen's d)
- Report exact p-values wherever possible alongside the summary statistics and 95% confidence intervals. These should be reported for all key questions and not only when the p-value is less than 0.05.

Statistical analysis, including tests of normality, are described in Materials and Methods “Statistical analysis” section (line 667), and in the text where appropriate.

Data from individual animals is shown whenever possible (Figure 3C; Figure 5; Figure 7C,E,G).

Exact p-values are reported wherever possible. Exact p-values are not reported for the non-significant results in Figure 4E due to the large number of days compared.

Please outline where this information can be found within the submission (e.g., page numbers or figure legends), or explain why this information doesn’t apply to your submission:

(For large datasets, or papers with a very large number of statistical tests, you may upload a single table file with tests, Ns, etc., with reference to page numbers in the manuscript.)

**Additional data files (“source data”)**

- We encourage you to upload relevant additional data files, such as numerical data that are represented as a graph in a figure, or as a summary table
- Where provided, these should be in the most useful format, and they can be uploaded as “Source data” files linked to a main figure or table
- Include model definition files including the full list of parameters used
- Include code used for data analysis (e.g., R, MatLab)
- Avoid stating that data files are “available upon request”

Please indicate the figures or tables for which source data files have been provided:

Code for dual-angle video-oculography and calibration of magnetic eye tracking system is available at <https://github.com/hpay/eyetrack>, as indicated in the Methods.
